# Effectiveness of bimanual kinesthetic teaching through different controller settings

**DOI:** 10.3389/fnbot.2026.1960197

**Published:** 2026-10-21

**Authors:** Oliver Avsec, Aleš Ude, Matjaž Mihelj, Andrej Gams

**Affiliations:** 1 Faculty of Electrical Engineering, University of Ljubljana, Ljubljana, Slovenia; 2 Department of Automatics, Biocybernetics and Robotics, Jožef Stefan Institute, Ljubljana, Slovenia

**Keywords:** bimanual robotics, dual-arm manipulation, human robot interaction (HRI), impedance control, kinesthetic teaching, KUKA LBR iiwa robot, robot guidance

## Abstract

Physical human-robot interaction (pHRI) through kinesthetic teaching enables intuitive robot programming, but extending this approach to bimanual systems introduces challenges in synchronized control. Thus, the best way to control bimanual robot systems for kinesthetic teaching remains unclear. In this paper we evaluate bimanual kinesthetic teaching from the demonstrator's perspective, showing how different modes of control can affect both demonstration performance and user experience. Our study, in which participants kinesthetically taught a bimanual robot to perform a pick-and-place task, showed that the relative stiffness of the two robots significantly affects task timing and operator perception.

## 1 Introduction

Transferring behavior and/or skills from a human to a robot, often referred to as learning by demonstration (LbD) or imitation learning (IL) (Billard et al., 2008; Schaal, 1999) can be done in many different ways. Novel means of tracking human movement have led, for example, to using virtual reality (VR) training systems, as shown by Maddipatla et al. (2025), or portable data collection devices without the need for physical robot hardware with visual feedback through augmented reality (AR) (Chen et al., 2025). Lately, specifically for whole-body motions, robot programmers/developers also use cameras (He et al., 2024). In the latter, feasible motions are picked using a privileged motion imitator, which already implies that the recorded motion needs to be adapted and changed in order to preserve correspondence (Mohammad and Nishida, 2013).

For manipulation tasks, kinesthetic teaching remains especially attractive because the demonstration is already adapted to the robot embodiment. It implicitly bypasses any potential correspondence problem (Mohammad and Nishida, 2013) and can directly encode contact-rich behavior (Argall et al., 2009). Kinesthetic teaching has also been shown to allow for highly intuitive skill transfer (Akgun et al., 2012). This was demonstrated, for example, in the peg in hole task by Abu-Dakka et al. (2015); Kramberger et al. (2017), where besides the motion also contact forces were transferred.

Numerous examples of kinesthetic teaching are reported in the literature, e.g., Caccavale et al. (2019). However, the literature is less generous on kinesthetically teaching bimanual robots. Bimanual kinesthetic teaching has been shown to be difficult because the demonstrator must coordinate two end-effectors, manage both absolute and relative motion, and cope with whatever coupling/compliance the controller imposes. That makes the controller setting a part of the interaction design, not just the low-level control implementation (Rakita et al., 2019).

In their paper on dual-arm robot box taping with kinesthetic teaching, Kim et al. (2016) show that high level planning for a specific task is necessary to operate the robot. Similarly, in a paper on kinesthetic teaching of bimanual tasks on a humanoid by Reinhart et al. (2012), the focus is on an architecture for flexible sequencing of such skills and not on the bimanual kinesthetic teaching itself. This raises the question of which robot control strategies are suitable for bimanual kinesthetic skill transfer and how they should be evaluated. In Suay et al. (2012), three different learning from demonstration approaches were qualitatively evaluated based on a survey. Quantitative assessments were also used, but kinesthetic teaching was not part of it. Similarly, for the case of adapting an already learned skill (i.e., coaching), both survey-based and quantitative evaluations were used by Gams and Ude (2016). In that work, visual and force feedback were used to modify motion under different compliance methods. Furthermore, a paper by Wrede et al. (2013) argues that at the time programming-by-demonstration approaches were not efficient for kinesthetic teaching of redundant robots and showed that typical teach-in procedures are too complex for novice users. A large user study based not only on subjective data from questionnaires but also objective data gathered directly from the live system was analyzed for significant effects regarding the test condition (assisted/non-assisted teach-in), showing that assisted gravity compensation mode has a positive impact on the usability and performance of programming trajectories on redundant robots.

Controller settings influence the demonstrator's interaction with both unimanual and bimanual robots; for instance, Wrede et al. (2013) found that assisted kinesthetic teaching of a redundant unimanual robot improved usability and trajectory-programming performance relative to non-assisted teaching. In bimanual teaching, an additional design choice concerns how responsibility for coordinating the two end-effectors is shared between the demonstrator and the controller. The present study investigates this choice for a rigid-object pick-and-place task by comparing different levels of relative coupling. These observations suggest that the controller setting should be regarded not only as a control-design choice but also as an interaction strategy that shapes the demonstrator's experience. Prior HRI research has shown that different interaction strategies can affect not only objective task performance, but also collaboration fluency, user preference, trust, workload, and overall user experience (Hoffman, 2019). Therefore, methods for bimanual kinesthetic teaching should be evaluated through a combination of quantitative task measures and operator-centered assessments.

To the best of our knowledge, prior work has not systematically evaluated bimanual kinesthetic teaching from the demonstrator's perspective, nor examined how different modes of control can affect both demonstration performance and user experience.

### 1.1 Contribution

As in the studies discussed above, we analyze robot learning from demonstration for bimanual kinesthetic teaching to determine preferred robot behavior from the demonstrator's perspective through subjective user assessments. A bimanual robot control setup that allows for decoupling of absolute and relative motion (Chiacchio and Chiaverini, 1998; Nemec et al., 2017) represents the core of the four teaching methods we evaluate. Its mathematical formulation is provided in the Appendix. The evaluated methods range from completely decoupled independent robot arms to strongly coupled bimanual behavior. We additionally evaluate which method enables the fastest and most accurate kinesthetic transfer of behavior to a bimanual robot. In this context, quantitative measures capture the efficiency and accuracy of demonstration, while operator-centered measures capture perceived workload, intuitiveness, and preference. A participant performing the kinesthetic learning by demonstration task in the experimental setup is depicted in Figure 1a and a better view of the task workspace is presented in Figure 1b.

**Figure 1:**
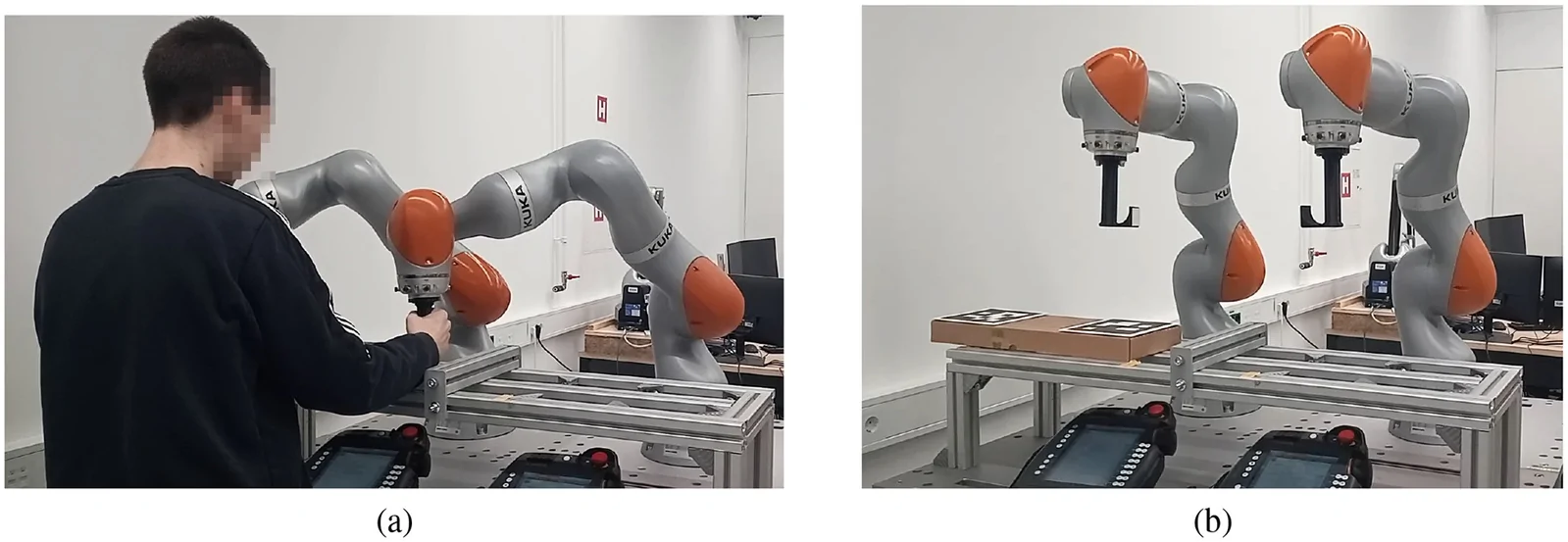
Experimental setup and task demonstration. **(a)** Participant guiding the robot end-effectors during the bimanual demonstration, where they were performing a kinesthetic teaching task on the dual-arm robotic platform (single frame from the demonstration; both end-effectors were held throughout the task). **(b)** Experiment setup with two KUKA LBR iiwa 14 robots, box with two ArUco markers and bench for pick-and-place task.

In the following, we first provide the design and procedures of our study in Section 2. The results of the study are presented in Section 3 and discussed in Section 4. A conclusion is given in Section 5. The underlying bimanual robot control is presented in the Appendix.

## 2 Study design

The study was designed to test whether increasing the relative stiffness between the two kinesthetically guided robots would (i) reduce the time needed to complete the pick-and-place task, (ii) improve the accuracy of object placement, (iii) produce a nonlinear change in the interaction forces, and (iv) lower the operator's subjective workload as measured by the NASA-TLX (Hart, 1988, 2006).

The study used a randomized within-subject design to compare the effectiveness and usability of four levels of coupling between the two robots, ranging from compliant individual robots to a highly coupled bimanual system. Coupling was achieved using a Cartesian Bimanual Impedance Controller, with settings determined empirically to ensure each level felt distinct while maintaining stability during contact.

### 2.1 Participants

The study included 15 volunteers (11 male, 4 female; mean age 23.8 ± 1.6 years). Their experience in robotics ranged from no previous experience to being robotics master's students. The subjects were informed about the experimental procedure, potential risks, and the aim of the study, and gave their written informed consent in accordance with the code for ethical conduct in research at Jožef Stefan Institute (JSI). This study was approved by the National Medical Ethics Committee (No.: 0120-228/2020-3).

For one participant, force recordings were unavailable across all four conditions due to a data-logging issue; NASA-TLX responses for this participant were retained. The force analysis in Section 3.3 is therefore based on *N* = 14.

### 2.2 Procedures

The experimental task involved coordinated bimanual manipulation of a rigid object. Participants performed a pick-and-place operation with a box (50.5 × 18 × 4.5 cm) equipped with two ArUco fiducial markers (Garrido-Jurado et al., 2014) of size 17 × 17 cm for optical tracking. The workspace included two target positions separated by a 6 cm high obstacle.

Participants demonstrated the task on a bimanual robot system through kinesthetic teaching under different bimanual impedance controller settings to assess the efficiency and quality of the learning process. The trajectory from picking up the box to placing it at the other target position was chosen freely by the subjects. All performance metrics were evaluated over the entire demonstration cycle, except for placing accuracy, which was assessed only during the placement phase.

During task execution opposing-side contact was required by the object and workspace geometry. Participants could choose their contact locations within that configuration; they were not choosing among different grasp configurations. Participants guided the two robots to grasp the box simultaneously, with one arm on each side, thereby, they were all the time physically guiding both robot end-effectors at the same time. Consequently, the relative-pose target enforced by the bimanual impedance controller was active throughout the entire demonstration cycle, including the grasping and placement phases, not only during transport. The desired relative pose (${\vec{p}}_{\text{rel},\text{d}}$, **R**_rel, d_) tracked by the relative impedance controller was a fixed reference, captured once at controller startup: the relative position reference was manually set to [0, 0.4, 0] m (x, y, z), with the relative orientation set to identity. It remained identical across trials. The slightly narrower distance of 40 cm is deliberately 10 cm narrower than the 50 cm length of the box, establishing an intentional pre-tension baseline to maintain continuous contact engagement. This reference was set precisely and repeatably before every trial; it therefore remained constant throughout the entire demonstration cycle and across all repetitions. Because the box is a rigid body, its geometry mechanically constrains the relative pose of the two end-effectors once both hands are in contact. This mechanical constraint was reinforced by the controller: the relative stiffness setting (**K**_rel_) determined how strongly the robots actively resisted deviations between the human-applied grasp and the controller's fixed relative-pose reference, providing an additional active mechanism for maintaining the relative pose on top of the passive constraint already imposed by the rigid box and grasp.

After completing the consent process, participants were guided through the experimental task without a preliminary practice or warm-up session with the bimanual system. This approach was chosen to capture the initial intuitiveness of the different controller settings during the first physical interaction. To reduce learning effects and fatigue, the order of the four settings was randomized for each participant using a computer-based random number generator. For each setting, participants were instructed to carry out the task 6 to 8 times, depending on whether they felt enough of their demonstrations were consistent. After each condition, participants provided subjective feedback on their perceived workload using the NASA-TLX questionnaire (Hart, 1988, 2006) and took a short rest of a few minutes before the next condition. Throughout the experiment, the system continuously logged joint states, interaction forces (estimated via inverse dynamics using joint torque sensor data), and task execution times for later analysis.

### 2.3 Measures

The robot's data acquisition was performed within the ROS 2 Humble environment (Macenski et al., 2022). Robot states, including joint positions and joint torques, were sampled at 500 Hz via the KUKA Fast Research Interface (FRI) (Schreiber et al., 2010). To reduce high-frequency noise, a causal exponential moving average filter with a time constant of τ = 0.006 s was applied online to the joint torque commands by the robot controller. A second causal moving average filter with a longer time constant of τ = 0.04 s was applied offline to the logged interaction forces, joint positions, and velocities. The short time constant preserved responsive, stable torque control online, while the longer offline filter was used solely to produce cleaner kinematic data for analysis and had no effect on the control loop. The time constants were selected empirically to balance noise rejection and signal responsiveness. External Cartesian wrenches were obtained from the controller's internal estimator, which uses joint-torque sensor measurements with compensation for link dynamics, gravity, and calibrated tool mass. Estimated wrenches were transformed into a global frame, with internal interaction forces calculated as the vector difference between the two arms, and overall loading summarized using the mean force magnitude across both end-effectors.

Other metrics that did not directly involve the robot were recorded on an external computer. The position of the manipulated object was tracked using an Intel RealSense™ D435 camera. Reference target poses (ref_1_, ref_2_) were established prior to the experiment by placing the box at the two desired target locations, recording their positions with the camera, and marking these locations in the workspace as a visual guide for the subject.

Placement accuracy was evaluated by using the planar (2D) Euclidean distance between the reference target coordinates and the object's final position. To ensure an unobstructed line of sight and prevent camera occlusion by the robotic arms or the participant's body, final pose logging was triggered only after the subject withdrew the robot arms, signaling satisfaction with the placement. Upon triggering, the object pose and system timestamp (*t*_*log*_) were recorded as the average marker position over 100 consecutive frames. Placement accuracy was evaluated in the horizontal plane only (x, y coordinates), since the target surface height was fixed and the vertical coordinate was therefore not informative for this measure. The box's planar position at each frame was computed as the average of the two ArUco markers' detected positions; because the robot arms were withdrawn before logging began, both markers were reliably visible throughout the 100-frame averaging window used to determine the final position. Task completion time *T*_*task*_ for each transport phase was calculated directly from these logged timestamps as: *T*_*task*_ = *t*_*log, destination*_−*t*_*log, origin*_, where *t*_*log, origin*_ is the timestamp recorded at the start target upon trial initiation and *t*_*log, destination*_ is the timestamp recorded at the destination target, when the robots started moving apart, i.e., started releasing the carried object. These timestamps were manually verified for all recordings. This protocol strictly captures the active manipulation and positioning phase while excluding setup intervals between trials.

To better assess the impact of different coupling settings on both performance metrics and the subjective experience of individuals demonstrating the task, we included a subjective workload metric measured by the NASA-TLX questionnaire. Subjective workload was quantified using the Raw NASA-TLX (Hart, 2006), calculated by averaging the scores from all subscales without participant-derived weighting, as pairwise weighting judgments were not collected in this study. Each category is measured on a 0-10 scale. Note that the Performance rating was transformed as 10 − original rating before calculating the overall Raw NASA-TLX score and presenting the subscale results. We report subscale-level results in addition to the overall score, since the coupling settings may redistribute workload across dimensions rather than uniformly reducing all subscales.

Statistical analysis was conducted to evaluate the influence of the four controller settings on demonstration performance, interaction forces, and subjective workload on subject-level means (*N* = 15 for performance and workload, *N* = 14 for force metrics). Data normality was assessed using the Shapiro-Wilk test (Shapiro and Wilk, 1965). For normally distributed metrics (object placement accuracy), differences across the four experimental conditions were evaluated using a one-way Repeated-Measures ANOVA. For non-normally distributed metrics (task completion time, interaction forces, and NASA-TLX workload scores), non-parametric comparisons across conditions were performed using the Friedman test (MacFarland and Yates, 2016).

For *post hoc* analysis, pairwise comparisons between settings (OFF, LOW, MID, HIGH) were conducted using the paired t-test for normally distributed data, and the Wilcoxon signed-rank test for non-normally distributed data. To control the family-wise error rate, *p*-values were adjusted using the Bonferroni correction (Miller, 2012; Bonferroni, 1936). The alpha level of significance was set *a priori* at 0.05. To quantify the practical significance of significant pairwise differences, we report the rank-biserial correlation coefficient *r* (for Wilcoxon tests) and Cohen's *d* (for paired t-tests), interpreted according to the standard thresholds proposed by Cohen (2013).

Statistical significance is reported at *p* < 0.05 (^*^), *p* < 0.01 (^**^), and *p* < 0.001 (^***^). All statistical analyses were performed using the SciPy library version 1.17 in Python.

### 2.4 Robot experimental setup

The experiment was conducted using two KUKA LBR iiwa 14 robot arms, which are collaborative robots with 7 degrees of freedom (DoF). The robots were controlled from a desktop PC that updated the joint positions and joint torques via the KUKA Fast Research Interface (FRI), which was then interfaced with ROS2 (Huber et al., 2023). The control algorithm was implemented in Python and updated the robot commands at 500 Hz for both robots.

Robot controllers operated in Joint Impedance Control mode (Schreiber et al., 2010) with joint stiffness set to 20 Nm/rad. The controller's internal joint stiffness is set very low, just enough to maintain position before the custom bimanual impedance controller takes over and asserts its own stiffness by commanding the corresponding joint torques. The effect of the internal impedance control is minimized by using lower internal stiffness and continuously updating the reference position. The different relative stiffness settings **K**_rel_ in the test are as follows:

- **OFF**: both robots are independent; all relative stiffness values are set to 0;
- **LOW**: robots are weakly coupled; translational relative stiffness values are set to 15 N/m and orientation relative stiffness to 4 N m/rad;
- **MID**: robots are decently coupled; translational relative stiffness values are set to 70 N/m and orientation relative stiffness to 10 N m/rad;
- **HIGH**: robots are strongly coupled; translational relative stiffness values are set to 200 N/m and orientation relative stiffnesses to 25 N m/rad.

The bimanual impedance controller computes the wrench based on the combined error vector (see Appendix for detailed definitions of absolute and relative errors, Jacobians, and the complete control framework)


$$\begin{array}{l} \vec{e}={\left[{\vec{e}}_{\text{rel}}^{⊤},{\vec{e}}_{\text{abs}}^{⊤}\right]}^{⊤}, \end{array}$$ (1)

$$\begin{array}{l} \vec{F}=\text{K}\vec{e}+\text{D}\overset{•}{\vec{e}}, \end{array}$$ (2)

where the stiffness and damping matrices are block-diagonal to decouple relative and absolute impedance control:


$$\begin{array}{l} \text{K}=\left[\begin{array}{cc} {\text{K}}_{\text{rel}} & 0\\ 0 & {\text{K}}_{\text{abs}} \end{array}\right],\;\text{D}=\left[\begin{array}{cc} {\text{D}}_{\text{rel}} & 0\\ 0 & {\text{D}}_{\text{abs}} \end{array}\right]. \end{array}$$ (3)

The relative stiffness values listed above populate the diagonal of **K**_rel_, while **K**_abs_ is set to very low values (1-3) to avoid interfering with the relative task. Damping gains are calculated from stiffness as $\text{D}=2\zeta \sqrt{\text{K}}$, where ζ is the damping ratio, set to ζ = 1 for relative and ζ = 0.5 for absolute damping. The higher relative damping reflects that relative coordination is the primary controlled dynamic. Absolute damping is set lower to avoid significantly influencing the relative task, while still providing some stability to the controller.

Since each of the robots has 7 DOF, a nullspace task was defined to control their postures. It was defined as impedance in the nullspace, where the target joint positions are set to the middle of their position range, in order to stay as far as possible from the joint position limits. The nullspace joint stiffness was set to 10 Nm/rad for all joints. The resulting wrench from the impedance law is transformed into joint torques and then added to the nullspace joint torques, which are forwarded to the robot's controller.

## 3 Results

In this section we review results from the experiment, in which the subjects had to kinesthetically demonstrate task execution on a bimanual robot system under different levels of coupling. The main metrics used to compare different settings were task completion time, object placement accuracy, internal interaction forces, and subjective workload score (NASA-TLX).

### 3.1 Task duration/completion time

Results for the time required to complete a single pick-and-place cycle are presented as a boxplot in Figure 2, where small gray dots indicate individual trial durations. A numerically detailed summary of these timing results, including mean and standard deviation, is provided in Table 1.

**Figure 2:**
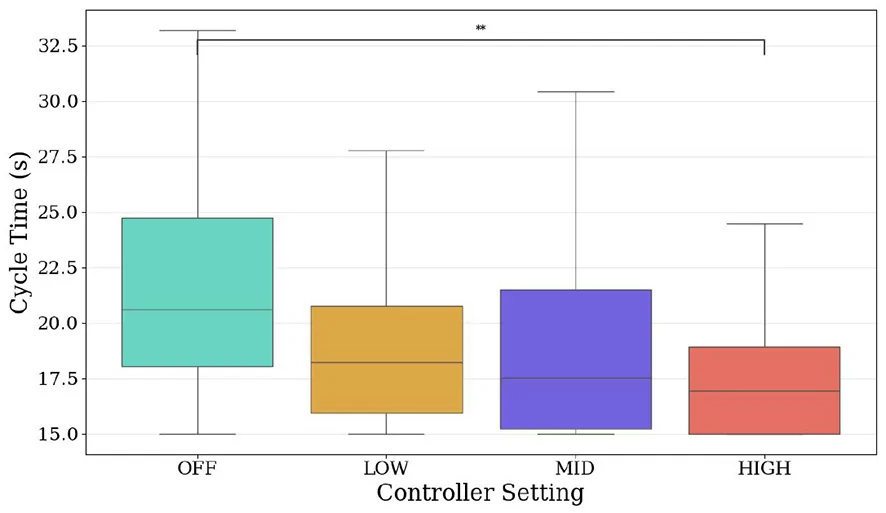
Task efficiency. Comparison of total cycle times (seconds) required to complete pick-and-place task for different bimanual impedance controller settings and *N* = 15 subjects.

**Table 1:** Task efficiency: cycle times across different bimanual impedance controller settings.

Controller setting	Mean (s)	SD (s)	Range (min–max) (s)
OFF	22.9	7.7	15.0–53.0
LOW	19.4	4.3	15.0–33.5
MID	20.0	7.1	15.0–57.6
HIGH	17.8	4.0	15.0–43.2

Statistical analysis revealed a significant effect of controller setting on task completion time (Friedman test: χ^2^(3) = 13.00, *p* = 4.64*e*−03, Kendall's *W* = 0.2889), indicating a moderate effect size.

Post-hoc pairwise comparisons with Bonferroni correction showed that the OFF condition differed significantly from the HIGH condition (*p* < 0.01, *r* = 0.93, rank-biserial correlation, large effect), with mean completion time decreasing from 22.9 s in OFF to 17.8 s in HIGH, a reduction of approximately 22.3%. Mean completion times for LOW (19.4 s) and MID (20.0 s) fell between these two extremes, though the pattern across all four settings was not strictly monotonic.

### 3.2 Placement accuracy

The calculated positioning errors for object placement across all four controller configurations are summarized in Figure 3. Mean results with standard deviation ranges are numerically listed in Table 2.

**Figure 3:**
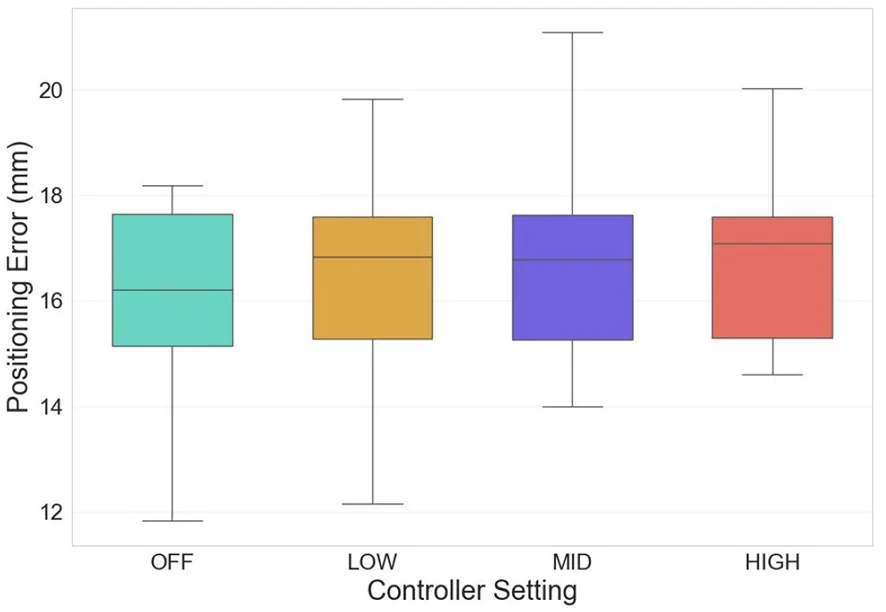
Placement accuracy. Comparison of final target positioning errors (mm) during pick-and-place task across different bimanual impedance controller settings for *N* = 15 subjects.

**Table 2:** Placement accuracy: Euclidean distance errors for the final object position.

Controller setting	Mean (mm)	SD (mm)	Range (min–max) (mm)
OFF	16.9	3.4	5.4–36.6
LOW	16.8	3.9	4.7–37.0
MID	16.1	5.2	1.7–35.4
HIGH	16.8	5.8	1.2–36.7

Placement accuracy was evaluated using a repeated-measures ANOVA on subject-level mean positioning errors (N=15). The analysis revealed no significant effect of controller setting on final placement error $\left[F_{\left(3,42\right)}=0.88,p=0.4616,\eta_{p}^{2}=0.0588\right]$, indicating a small effect size.

Post-hoc pairwise comparisons with Bonferroni correction confirmed no statistically significant differences between any controller settings (all *p*>0.05). No statistically significant between-condition difference in final placement error was detected. Compensatory slowing by participants may have reduced sensitivity to controller-related differences, but this explanation was not tested separately.

### 3.3 Interaction forces

Figure 4 shows the mean force magnitude summed across both robots' end-effectors, where only forces during the carrying process were taken into account. As described in Section 2.2, the relative-pose reference tracked by the impedance controller is set deliberately narrower than the box, establishing a fixed pre-tension against the box's rigid geometry once both end-effectors are in contact. By design, this means the controller actively maintains a compressive load whose magnitude scales with *K*_rel_, and this is expected to contribute mechanically to interaction force across settings, consistent with our original hypothesis that coupling strength would produce a change in interaction forces (Section 2). This raises the question of how much of the observed force increase reflects this designed-in mechanical contribution versus operator behavior or other unmodeled dynamics. We address this directly below via the commanded/residual decomposition, rather than assuming that any particular force measure is unaffected by the controller by construction.

**Figure 4:**
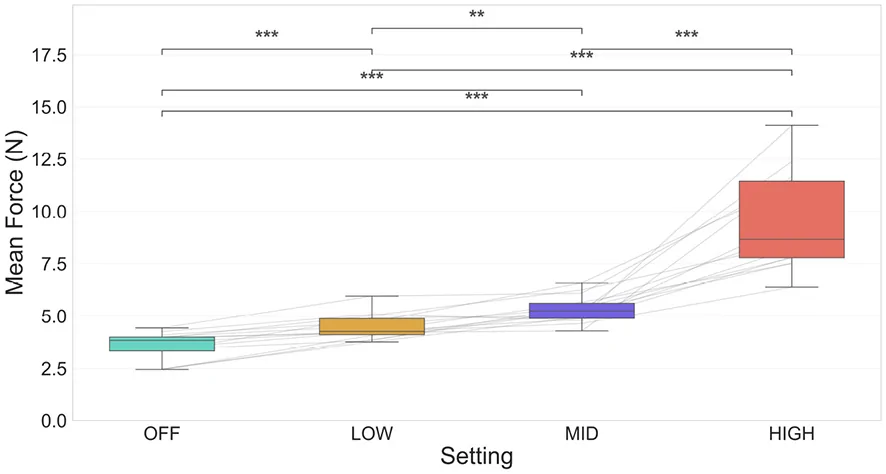
Mean force magnitude summed across both end-effectors, by controller setting. Gray lines connect each participant's values across settings. This measure approximates the combined physical engagement of the operator and the robots with the task.

We report the summed force across both arms, rather than the relative (differential) wrench, as our primary interaction-force measure, since it also captures the operator's own applied forces on each arm individually, rather than only their net effect on relative coordination.

Summed force increased monotonically with coupling strength. Statistical analysis, restricted to the *N* = 14 participants with complete force recordings across all four conditions (Section 2.1), revealed a significant effect of controller setting on summed force magnitude (Global Friedman test: *Q*(3) = 31.71, *p* = 6.01*e*−07, Kendall's *W* = 0.7551, indicating a very large effect). Post-hoc pairwise comparisons with Bonferroni correction (Table 3) showed significant differences between all six setting pairs, with the weakest, though still significant, distinction between LOW and MID (*p*_adj_ = 0.005, *r* = 0.959) and the largest, most pronounced increase occurring between MID and HIGH.

**Table 3:** *Post-hoc* pairwise comparisons of mean summed force across both end-effectors (Wilcoxon signed-rank test, Bonferroni-corrected; *N* = 14).

Comparison	*W*	*p*_adj_	Sig.	*r*
OFF vs. LOW	0.0	< 0.001	^***^	1.000
OFF vs. MID	0.0	< 0.001	^***^	1.000
OFF vs. HIGH	0.0	< 0.001	^***^	1.000
LOW vs. MID	4.0	0.005	^**^	0.959
LOW vs. HIGH	0.0	< 0.001	^***^	1.000
MID vs. HIGH	0.0	< 0.001	^***^	1.000

To better understand what this increase in force reflects, we decomposed the measured relative wrench into a controller-attributable component (the commanded wrench, computed from the relative position and velocity error and the corresponding stiffness and damping gains) and a residual component, obtained as the vector difference between the measured and commanded wrench (Figure 5). This addresses a related question: whether the increase in interaction force with coupling strength simply reflects the controller exerting a larger commanded force, independent of operator behavior – which would make the LOW–MID–HIGH comparison largely uninformative about the human contribution.

**Figure 5:**
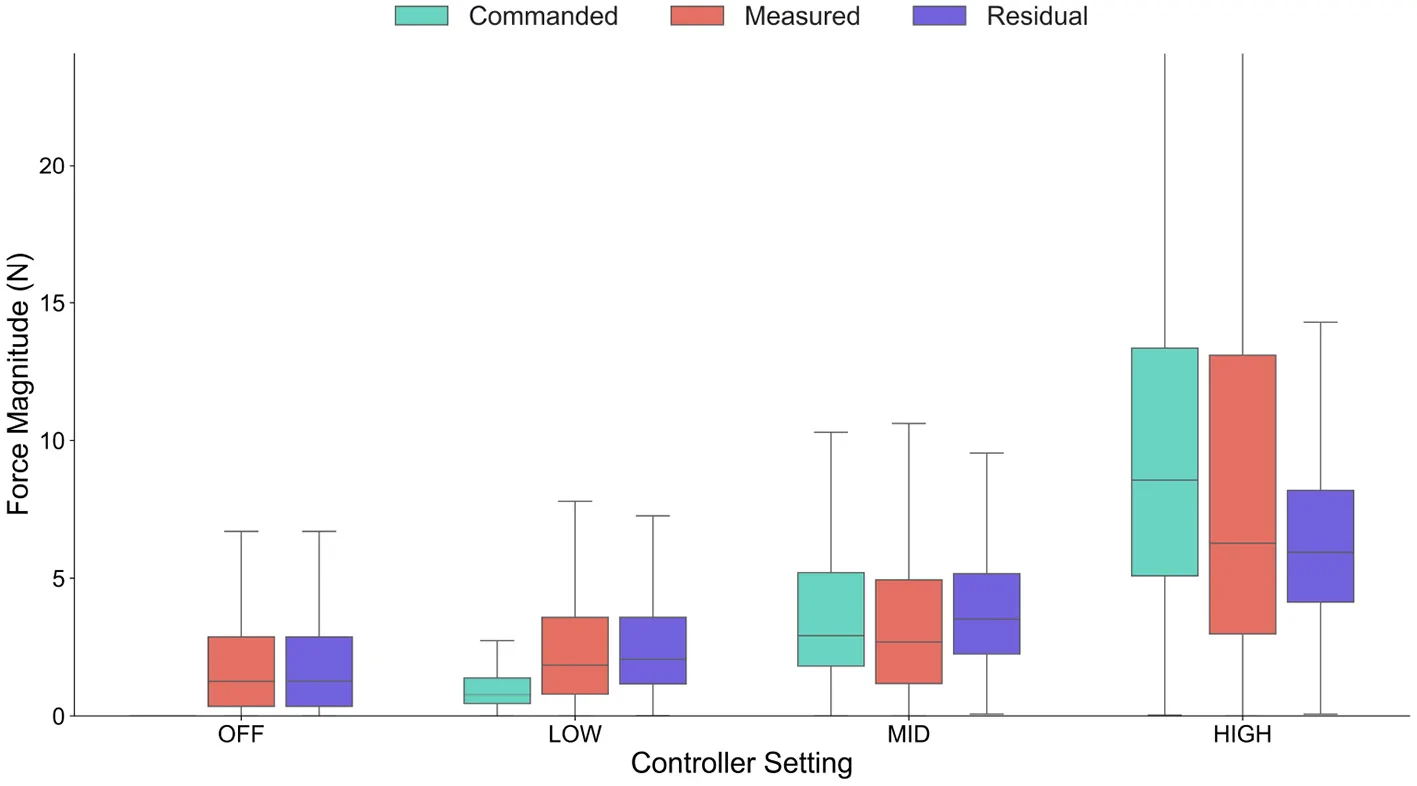
Decomposition of the relative interaction wrench into a controller-attributable component (Commanded), the measured wrench (Measured), and the residual (the vector difference between measured and commanded), by controller setting.

The commanded wrench explained an increasing share of the measured wrench's variance with coupling strength (linear-regression *R*^2^, median = 0.01, 0.09, and 0.37 for LOW, MID, and HIGH, respectively), consistent with the controller's growing role at higher coupling. Critically, however, the residual component also increased steadily in absolute terms across conditions (median 1.3, 2.1, 3.6, and 6.0 N for OFF, LOW, MID, and HIGH, respectively). If the interaction-force increase were fully attributable to the controller's own commanded response, this residual would be expected to remain approximately constant across settings, reflecting a fixed baseline of unmodeled or operator-driven force. Its continued growth indicates that factors beyond the modeled controller response also contribute to the observed increase in interaction force, and that the LOW–MID–HIGH comparison is not reducible to the controller's commanded reference alone. We note that this residual cannot be attributed to the operator in isolation, as it also absorbs any unmodeled controller/actuation dynamics not captured by our commanded-wrench model, which does not include the wrench-to-torque transform or associated torque-limit clipping.

We note that this decomposition is applied to the relative wrench specifically, which is the quantity most directly shaped by the controller's deliberate pre-tension; it therefore represents a conservative test of whether the observed force increase can be explained by the intentional coupling design alone. Even under this stringent test, the residual component's continued growth across conditions indicates that the designed-in pre-tension does not fully account for the observed increase in interaction force, and that the summed-force comparison retains information beyond this mechanical design choice.

### 3.4 Subjective workload

Results for the average subjective workload score for the task investigated in this study performed with different settings are presented in Figure 6. The largest differences were observed between the OFF and HIGH settings but also between OFF and LOW settings (*p* < 0.01). Statistically significant differences (*p* < 0.05) were found between the OFF and MID settings. The average subjective workload shows a trend where a higher stiffness setting results in a lower (better) score, although differences between higher settings were not statistically significant.

**Figure 6:**
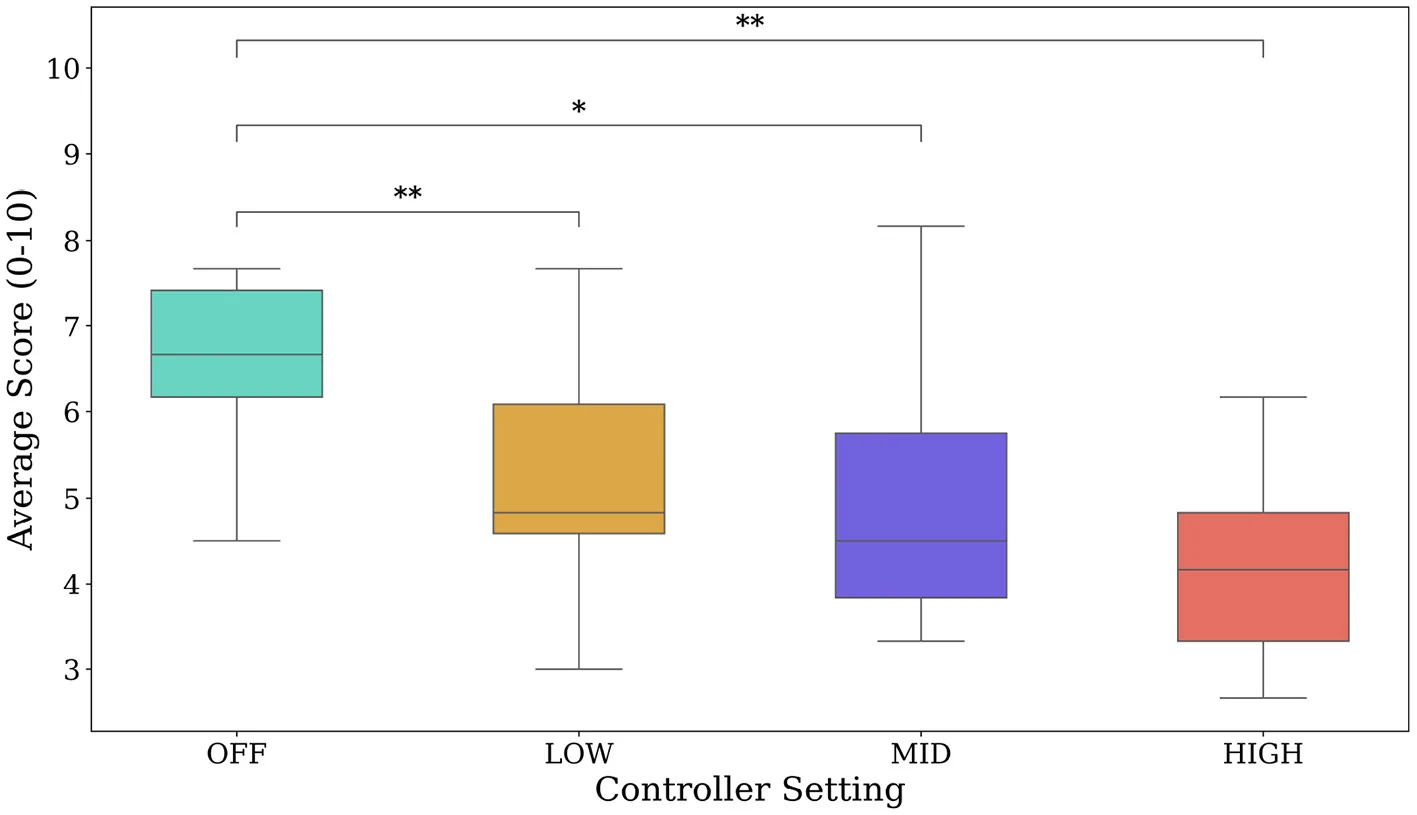
Overall subjective workload. Comparison of average NASA-TLX scores for pick-and-place task under varying controller stiffness parameters. Gray dots represent individual data points.

Additionally, average scores for each subscale are presented in Figure 7. Generally, most of subscale scores improve with higher relative stiffness settings.

**Figure 7:**
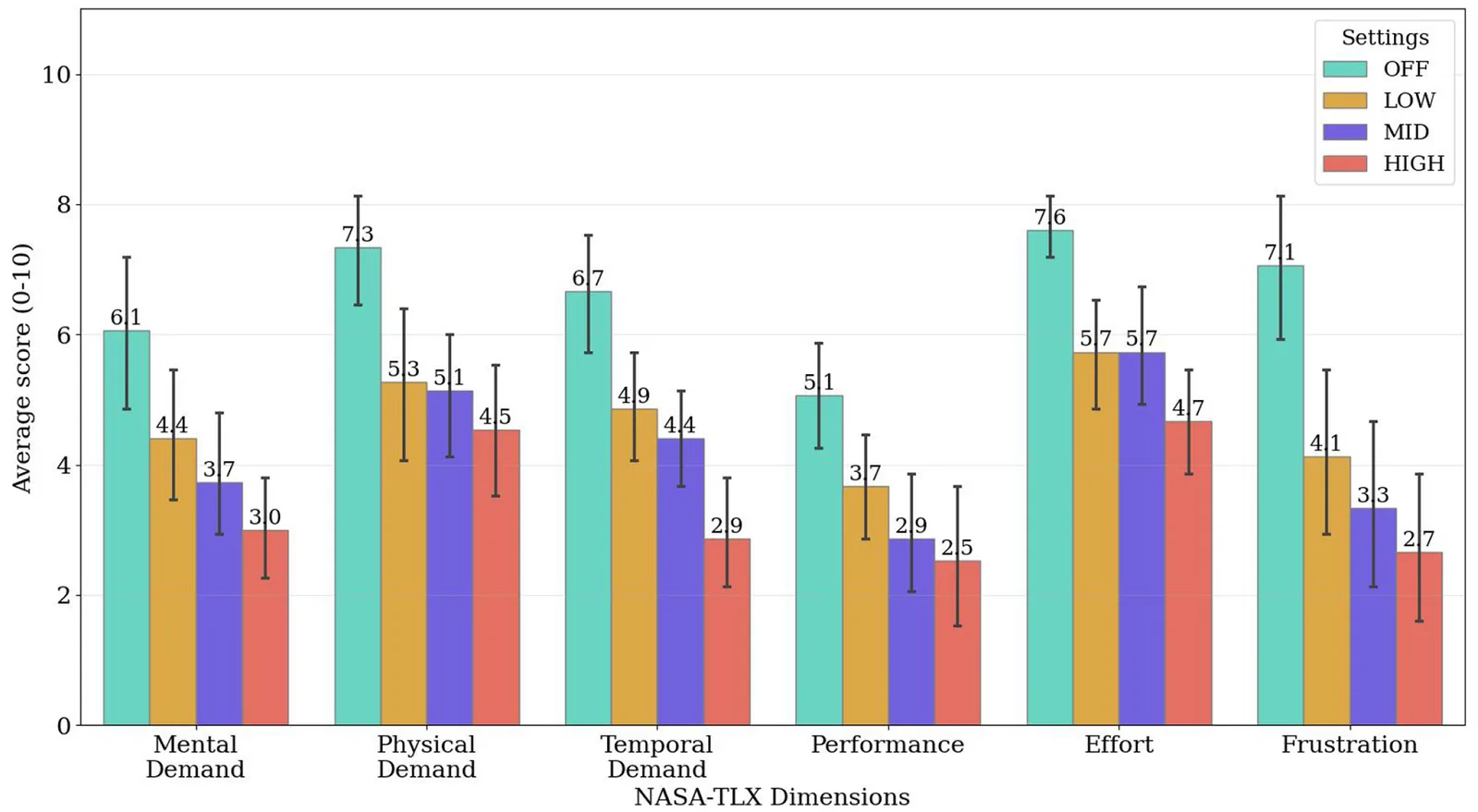
NASA-TLX sub-scale ratings. Comparison of the six workload dimensions for pick-and-place task across four bimanual impedance controller settings.

## 4 Discussion

The results show that the controller relative stiffness setting influenced the time required to complete one cycle of the task. As the level of relative stiffness (K_rel_) increased, the average time required for the pick-and-place task decreased. The most significant differences were observed between the OFF and HIGH settings. When participants used the setting in which both robots were decoupled, they had more difficulty coordinating the robots, resulting in the longest average cycle times observed in the trial. Consistency for this setting was also the lowest, meaning some participants had little difficulty, while others required much more time and even had to repeat the cycle multiple times due to dropping the object.

Accuracy remained largely consistent across all four conditions. Although higher stiffness settings (MID and HIGH) provided better coordination during the transfer phase, they did not necessarily result in more precise final placement than the OFF setting. We attribute this to the fact that, regardless of the controller, participants consistently performed a deliberate slow-down phase before releasing the object. This suggests that the human operator's precision, rather than the level of robotic assistance, was the primary factor in the final placement phase. In accordance with Fitts' Law (Fitts, 1954), while robotic assistance can reduce movement effort during gross transport, the terminal fine-positioning phase remains bottlenecked by human sensorimotor precision. This introduces a natural lower bound on total cycle time, explaining why higher assistance levels yield diminishing returns in overall task duration.

Results in Figure 4 show how the summed interaction force across both end-effectors varies with relative stiffness. The figure indicates that force magnitude increases monotonically from the uncoupled setting (OFF) to the strongly coupled setting (HIGH), with the most pronounced increase occurring between MID and HIGH.

This finding is, at first glance, in tension with the subjective workload results (Figure 7), where higher coupling settings were consistently rated as less physically and mentally demanding despite requiring more force. We attribute this dissociation to several contributing factors rather than a single cause. At lower coupling settings, the operator must actively coordinate both arms to maintain the correct relative pose – increasing the relative stiffness effectively shifts the operator's task from this relative task, which demands sustained, fine-grained corrective force to avoid deviating from the desired relative pose, to a more absolute task, where the controller manages relative coordination and the operator only needs to guide the system to the target pick-up and placement locations. Part of the force increase is also mechanically expected rather than behavioral: as established in Section 3.3, the controller's deliberate pre-tension against the box means a portion of the rising force reflects the fixed, designed-in compressive load scaling with *K*_rel_, independent of the operator's own actions. Since this component is not generated by the human, it is unsurprising that it does not register as increased perceived effort. Although this shift involves applying larger raw forces at higher settings, it removes the penalty for imprecision, so the increase in force is not perceived as more effortful. This interpretation is consistent with the NASA-TLX results, where higher stiffness settings consistently reduced perceived mental and physical demand, and with participants' free-text feedback, where difficulty at the OFF setting was attributed partly to the smooth, round handle making orientation control especially demanding without coupling assistance. Part of the residual difference may also reflect unmodeled controller and interaction dynamics not captured by our commanded-wrench model (see Section 3.3).

We note that even the highest forces observed in this study remain modest in absolute terms, and this dissociation should not be extrapolated beyond the tested range. As discussed below, a subset of participants preferred the MID setting over HIGH, suggesting that the practical benefit of increased coupling may plateau, or even reverse, beyond a certain threshold rather than increasing indefinitely.

Results for perceived workload (Figure 6) show that, in general, higher relative stiffness settings result in better scores for the pick-and-place task. Even when examining individual TLX subscale ratings (Figure 7), it is evident that, among the settings used, higher relative stiffness improved or at least maintained the score compared to lower settings.

Some participants mentioned that the pick-and-place task was especially difficult with the OFF setting, partly because the handle was round and relatively smooth. As a result, maintaining tool orientation in the OFF setting was challenging. A smaller portion of participants also noted that they felt the MID setting was much better than the HIGH setting, which could be explained by the fact that the practical limit for high stiffness may vary between individuals. This could be improved by using individually selected or adaptive gains, since in this survey the gains were set empirically.

Beyond the specific benefits observed for this task, our findings raise a more general design question for bimanual kinesthetic teaching: which aspects of a task should remain under the demonstrator's direct control, and which constraints are better enforced by the controller? In our task, the relative pose between the two end-effectors was intended to stay fixed throughout the entire demonstration cycle, since the box is rigid and its geometry does not change; a high, constant relative stiffness could therefore support this constraint continuously without ever working against the demonstrator's intent. This is not true of every bimanual task. Tasks that require the demonstrator to deliberately change the relative pose during the demonstration, for example, changing the relative orientation of two separately held parts during assembly or manipulating a deformable object whose effective relative pose between grasp points is not constant, would place a high, fixed relative stiffness in direct opposition to the intended motion at precisely the moments it needs to change. Similarly, asymmetric bimanual tasks, where the two arms play functionally distinct roles (e.g., one arm stabilizing an object while the other manipulates it), may call for a fundamentally different coupling structure than the symmetric, constant relative-pose assistance evaluated here. Our results support benefits of relative coupling in the rigid-object pick-and-place task studied here. They suggest possible applicability to other tasks with fixed relative-pose constraints, but that extension requires experimental validation.

It is also worth distinguishing between the demonstration performance evaluated here and the quality of any autonomous behavior subsequently learned from these demonstrations. Our measures—task completion time, placement accuracy, interaction force, and subjective workload—characterize the demonstration process itself, i.e., how effectively and comfortably the operator could kinesthetically guide the robot through the task. They do not directly assess whether demonstrations collected under different coupling settings differ in their suitability as training data for a subsequently learned policy (e.g., via learning from demonstration or imitation learning). It is possible, for instance, that a coupling setting which eases the demonstration process by rigidly enforcing the relative-pose constraint also reduces the natural variability in the human's applied force and hand placement that might otherwise inform a more robust or generalizable learned policy. Evaluating this downstream effect was beyond the scope of the present study, which focused on the demonstrator's experience during teaching itself, and is an important direction for future work.

Several limitations of this study should be acknowledged. The study did not include a dedicated practice or warm-up phase prior to data collection. Although the order of coupling settings was randomized across subjects, the limited sample size (*n* = 15) means this randomization may not fully distribute potential learning or fatigue effects across conditions. This sample is relatively small and demographically homogeneous, and may not capture the range of physical characteristics, prior manual-handling experience, or task-relevant intuition present in a real-world industrial workforce. Some of the observed between-condition differences may therefore be partially confounded by intra-session learning. Suboptimal camera placement likely added noise to placement accuracy measurements. The findings are currently limited to a single pick-and-place task with a rigid object; generalization to other bimanual tasks requires further validation. Fixed stiffness gains did not account for individual preferences, and the not large sample size (*n* = 15) somewhat limits generalizability of subjective measures. Because operators consistently slowed their motion prior to releasing the object, they were likely able to compensate for any placement-accuracy differences introduced by the coupling setting. This behavior likely masks any true effect of the controller on placement precision; accordingly, the absence of a significant difference in placement accuracy across conditions should not be interpreted as evidence that the controller has no effect on precision, but rather reflects a limitation of this task design in isolating that effect. Additionally, the observed dissociation between objective interaction force and subjective effort should not be extrapolated beyond the force range tested here; it remains possible that sufficiently high coupling and force levels would eventually increase perceived effort despite improved coordination, consistent with some participants' preference for the MID over the HIGH setting. Future work should address these limitations through adaptive control, broader task sets, larger participant pools and allow a dedicated practice phase to stabilize the performance metric. Furthermore, although the study recorded complete directional force vectors and individual arm trajectories for both robots, a deeper analysis of interaction strategies—such as distinguishing between push and pull dynamics during bimanual guidance—was beyond the scope of this work and is left for future investigation.

## 5 Conclusion

The main conclusion is that assistance with relative coordination, implemented here using a bimanual impedance controller, leads to: (i) faster kinesthetic teaching of the bimanual robot; (ii) no statistically significant difference in measured final placement error on pick-and-place task accuracy from different relative stiffness settings; and (iii) & (iv) although the total force exerted between the bimanual system and the human increased with relative stiffness, this was not reflected in subjective effort: users consistently rated higher settings as less demanding and still preferred them, describing them as “less taxing to use” and “improving performance” for a pick and place task. However, relative stiffness settings should feel sufficiently stiff while still maintaining stability.

Thus, we can conclude that, for this pick-and-place task, sufficiently high relative stiffness between the two robots yields the clearest overall improvement over fully independent (OFF) control, reducing task completion time and consistently improving subjective workload without a measurable cost to placement accuracy. Lower coupling levels (LOW, MID) also significantly reduced subjective workload relative to OFF, but did not yield a comparably clear objective benefit in task timing. It is therefore only when considering both objective and subjective measures jointly that the HIGH setting stands out as substantially different from fully independent control; our results do not support the broader claim that any form of relative coordination is preferable to independent control, since this holds unambiguously only for sufficiently high relative stiffness. This is broadly in line with (Ganesh et al. 2014), who showed that physical assistance can improve motor performance, though our data suggest that a clear, robust benefit in this task requires sufficiently strong coupling rather than coordination of any strength.

Another conclusion is that coordination should be tailored to the specific task. Thus, future work will include adaptive stiffness for the bimanual system, expanded testing across a wider range of tasks—including asymmetric and deformable-object manipulation—and an evaluation of how the coupling setting used during demonstration affects the quality of autonomous behavior subsequently learned from those demonstrations.

## Data Availability

The raw data supporting the conclusions of this article will be made available by the authors, without undue reservation.
