# Supplementary material for: Effectiveness of bimanual kinesthetic teaching through different controller settings
Source: Front Neurorobot. 2026 Oct 21;20:1960197. doi: 10.3389/fnbot.2026.1960197 (PMC13731373; doi:10.3389/fnbot.2026.1960197)
Supplement: Supplementary file 1 [file Supplementary_file_1.pdf]

Supplementary Material

S1 Bimanual Robot Control

This supplementary material describes the basic definitions of the relative and absolute workspace of a bimanual robot. For a more comprehensive overview of bimanual workspace, see Chiacchio et al. (1996).

In our experiments, we used only symmetric bimanual control, though this does not have much impact since only the absolute workspace differs between symmetric and asymmetric control, and the absolute stiffness was consistently very low or zero. The absolute pose describes the position and orientation of a common coordinate frame of the robots, expressed from the viewpoint of the inertial coordinate frame Eq. (S1)

$$\vec{p}_{\mathrm{abs}} = \frac{1}{2}(\vec{p}_1 + \vec{p}_2), \quad \mathbf{R}_{\mathrm{abs}} = \mathbf{R}_1 \exp\left(\frac{1}{2}\log(\mathbf{R}_1^\top \mathbf{R}_2)\right), \tag{S1}$$

where matrix exponential and its inverse, the logarithm, are respectively defined as

$$\exp(\vec{r}) = \begin{cases} \mathbf{I}, & \vec{r} = 0 \\ \mathbf{I} + \sin(\|\vec{r}\|)\dfrac{[\vec{r}]_\times}{\|\vec{r}\|} + (1 - \cos(\vec{r}))\dfrac{[\vec{r}]_\times^2}{\|\vec{r}\|^2}, & \text{otherwise} \end{cases}, \tag{S2}$$

$$[\vec{r}]_\times = \begin{bmatrix} 0 & -r_z & r_y \\ r_z & 0 & -r_x \\ -r_y & r_x & 0 \end{bmatrix}.$$

and

$$\log(\mathbf{R}) = \begin{cases} [0,0,0]^\top, & \mathbf{R} = \mathbf{I} \\ \theta\vec{n}, & \text{otherwise} \end{cases}, \tag{S3}$$

$$\theta = \arccos\left(\frac{\mathrm{trace}(\mathbf{R}) - 1}{2}\right), \ \vec{n} = \frac{1}{2\sin(\theta)}\begin{bmatrix} r_{32} - r_{23} \\ r_{13} - r_{31} \\ r_{21} - r_{12} \end{bmatrix}.$$

The relative pose describes the position and orientation of one robot TCP relative to the other Eq. (S4)

$$\vec{p}_{\mathrm{rel}} = \vec{p}_2 - \vec{p}_1, \quad \mathbf{R}_{\mathrm{rel}} = \mathbf{R}_1^\top \mathbf{R}_2. \tag{S4}$$

Then twists for absolute and relative space are defined as

$$\vec{\xi}_{\mathrm{abs}} = \frac{1}{2}\begin{bmatrix} \mathbf{I}_6 & \mathbf{I}_6 \end{bmatrix}\begin{bmatrix} \vec{\xi}_1 \\ \vec{\xi}_2 \end{bmatrix}, \quad \vec{\xi}_{\mathrm{rel}} = \vec{\xi}_2 - \vec{\xi}_1. \tag{S5}$$

Jacobians for absolute and relative space are defined as

$$\mathbf{J}_{\text{abs}} = \frac{1}{2}\begin{bmatrix}\mathbf{J}_1 & \mathbf{J}_2\end{bmatrix}, \quad \mathbf{J}_{\text{rel}} = \begin{bmatrix}-\mathbf{J}_1 & \mathbf{J}_2\end{bmatrix}, \tag{S6}$$

and then for convenience stacked as

$$\mathbf{J} = \begin{bmatrix}\mathbf{J}_{\text{rel}} \\ \mathbf{J}_{\text{abs}}\end{bmatrix}. \tag{S7}$$

Lastly needed for impedance controller are errors in absolute and relative coordinates

$$\vec{e}_{\text{abs}} = \begin{bmatrix}\vec{p}_{\text{abs,d}} - \vec{p}_{\text{abs}} \\ \log\left(\mathbf{R}_{\text{abs,d}}\mathbf{R}_{\text{abs}}^{\top}\right)\end{bmatrix}, \quad \vec{e}_{\text{rel}} = \begin{bmatrix}\mathbf{R}_1\vec{p}^{\,1}_{\text{rel,d}} - \vec{p}_{\text{rel}} \\ \log\left(\mathbf{R}_{\text{rel,d}}\mathbf{R}_{\text{rel}}^{\top}\right)\end{bmatrix}, \tag{S8}$$

where the subscript $_d$ denotes a desired quantity, and the superscript ^{1} indicates the vector is expressed in robot 1's end-effector frame and mapped to the world frame via $\mathbf{R}_1$. Relative task error $\vec{e}_{\text{rel}}$ is expressed in the global frame. Errors in both coordinates are combined like in Eq. S7

$$\vec{e} = \begin{bmatrix}\vec{e}_{\text{rel}} \\ \vec{e}_{\text{abs}}\end{bmatrix}. \tag{S9}$$

Then to compute error wrenches the following equation is used

$$\vec{F} = \mathbf{K}\,\vec{e} + \mathbf{D}\,\dot{\vec{e}}, \tag{S10}$$

where $\mathbf{K}$ and $\mathbf{D}$ are gain matrices for stiffness and damping. Finally, the wrench is converted by transposed Jacobian matrix into joint torques and sent to the control interface

$$\vec{\tau} = \mathbf{J}^{\top}\vec{F}. \tag{S11}$$

References

Chiacchio, P., Chiaverini, S., and Siciliano, B. (1996). Direct and inverse kinematics for coordinated motion tasks of a two-manipulator system. *J. Dyn. Syst. Meas. Control* 118, 691–697. doi: 10.1115/1.2802344.
